# Missing information in birth certificates in Brussels after reinforcement of data collection, and variation according to immigration status. A population-based study

**DOI:** 10.1186/0778-7367-70-25

**Published:** 2012-11-08

**Authors:** Anne-Frederique Minsart, Pierre Buekens, Myriam De Spiegelaere, Sabine Van de Putte, Virginie Van Leeuw, Yvon Englert

**Affiliations:** 1 Perinatal Epidemiology Center ‘CEpiP’ School of Public Health, University Hospital Erasme and Faculty of Medicine, Free University of Brussels, Brussels, 1070, Belgium; 2 Department of Obstetrics and Gynecology and Research Laboratory for Human Reproduction, University Hospital Erasme and Faculty of Medicine, Free University of Brussels, Route de Lennik 808, Anderlecht, Brussels, 1070, Belgium; 3 School of Public Health and Tropical Medicine, Tulane University, New Orleans, LA, USA; 4 Brussels-Capital Health and Social Observatory, Brussels, Belgium

**Keywords:** Birth certificates, Validation studies, Bias, Epidemiologic

## Abstract

**Background:**

A problem repeatedly reported in birth certificate data is the presence of missing data. In 2008, a Centre for Perinatal Epidemiology was created inter alia to assist the Health Departments of Brussels-Capital City Region to check birth certificates. The purpose of this study is to assess the changes brought by the Centre in terms of completeness of data registration for the entire population and according to immigration status.

**Methods:**

Birth certificates from the birth registry of 2008 and 2009 of Brussels were considered. We evaluated the initial missing information in January 2008 (baseline situation) and the corresponding rate at the end of 2008 after oral and written information had been given to the city civil servants and health providers. The data were evaluated again at the end of 2009 where no reinforcement rules were adopted. We also measured residual missing data after correction in socio-economic and medical data, for the entire population and according to maternal nationality of origin. Changes in registration of stillbirths were estimated by comparison to 2007 baseline data, and all multiple births were checked for complete identification of pairs.

**Results:**

Missing information initially accounted for 64.0%, 20.8% and 19.5% of certificates in January 2008, December 2008, and 2009 respectively. After correction with lists sent back to the hospitals or city offices, the mean residual missing data rate was 2.1% in 2008 and 0.8% in 2009. Education level and employment status were missing more often in immigrant mothers compared to Belgian natives both in 2008 and 2009. Mothers from Sub-Saharan Africa had the highest missing rate of socio-economic data. The stillbirth rate increased from 4.6 ‰ in 2007 to 8.2 ‰ in 2009. All twin pairs were identified, but early loss of a co-twin before 22 weeks was rarely reported.

**Conclusions:**

Reinforcement of data collection was associated with a decrease of missing information. The residual missing data rate was very low. The stillbirth rate was also improved but the early loss of a co-twin before 22 weeks seems to remain underreported.

## Background

Birth certificate data provide key information for caregivers, researchers, and policy makers to evaluate trends in pregnancy outcomes and in care being delivered to pregnant women on a population basis.

Little is known about how the completeness of birth certificate data varies by characteristics of the mother. Missing data have been found repeatedly in some circumstances, such as maternal social deprivation and low birthweight, and this suggests that the completeness may not be uniform across subgroups of the population of women [1]. Few studies have concentrated on missing data in immigrant subgroups [2].

Another problem reported is the registration of stillbirths, as registration rules and practices may vary among countries [3,4] and maternity wards within the same country [3]. Many fetal deaths between 20 and 28 weeks might go unreported [5,6]. Stillbirth prevention is closely linked with prevention of neonatal deaths, and knowledge of causes could contribute to find solutions for prevention [7]. Besides the assessment of individual live and stillbirth certificates, methodological management of multiple births certificates is of great importance. Birth and death certificates classify individuals as twins or higher order multiples, but do not identify pairs [8].

More studies on efforts to implement continuous quality improvement initiatives are required [9,10]. It has been suggested that reliability of birth certificate data may be facilitated by education of medical and non-medical personnel involved in the birth certificate data collection and by the use of electronic systems to edit inconsistencies in the data prior to record acceptance as well as feedback to hospitals on reported data [11]. In 2008, a Centre for Perinatal Epidemiology was raised inter alia to assist the Health Departments of the Brussels Capital City Region to gather and check birth certificates and give feedback to maternity wards and city offices. The purpose of this article is to assess the changes brought by the Centre regarding completeness of data, taking into account the immigrant status of the mother.

## Methods

The birth register includes birth certificates of all live births and stillbirths from 500 grams or 22 weeks’ gestation of the 13 maternities of Brussels. The coverage of the register is also including births to women staying in Belgium illegally and asylum seekers and planned and unplanned home births. Totally, 23,375 live births and 197 stillbirths were registered in 2008, 24,157 and 226 respectively in 2009.

Neonatal, maternal, socio-economic, and medical forms are automatically linked with an identical file number. Hospitals and health workers are responsible for reporting all stillborn and live born babies on dedicated paper sheets. Medical data are registered by midwives and gynaecologists implicated in the index birth, and socio-economic data are filled with the parents at the civil registration service within 15 days of the birth. The register is anonymous and publicly available, and was accessed with permission from the Commission for the Protection of Privacy, the Brussels Health Observatory and the Health Department of the French Community of Belgium.

The Centre for Perinatal Epidemiology is ran by midwives, gynaecologists, epidemiologists, and paediatricians, and it is supervised by epidemiologists from the Health Departments of Brussels-Capital city region. The Centre has implemented several procedures intending to inform caregivers about the necessity to produce accurate data. Postal reminders were sent to all gynaecologists and midwives in 2008 with definition of each item figuring on the birth certificate, and chief-midwives were all met once a month in 2008 to ensure follow-up. Clear recommendations regarding the mandatory registration of stillbirths after 22 weeks of gestation or weighing ≥500 g were made. Maternity wards were asked to notify if an early loss (before 22 weeks) of one twin had occurred in a multiple gestation. City civil servants received written information and recommendations regarding socio-demographic data registration. Attendants of the Centre were reachable by means of telephone calls for all questions regarding the certificates. Trained administrative workers encode social and medical characteristics from all live births occurring in Brussels, except births from two maternities whose medical data are encoded by the Flemish Community, accounting for 3694 births in 2008 and 2343 in 2009. After encoding data, a monthly visual check of all variables is performed to detect typing errors. Seemingly incorrect information (extreme or implausible values with likelihood of being data errors) or missing data are also routinely detected by the use of electronic systems and an error listing is sent back to the hospitals or city offices for correction.

Multiple gestations were ascertained on the basis of the notification of a multiple pregnancy by health workers on the birth certificate, coupled with infant’s birth date, parents’ birth date and socio-economic characteristics, delivery site, gestational age, and pregnancy complications. If a difference was noted, additional information was required from the maternity. Similar infant’s and parents’ birth date were also checked to exclude missing notification of a twin birth.

Stillbirth certificates are managed differently as these certificates are encoded by trained nurses in another regional Centre, sent back electronically to our database, and checked on a yearly basis by our Centre.

All data are analyzed and an annual individual perinatal report is sent back to maternity wards and city offices together with a full report including all deliveries in the city.

Data assessment and quality improvement are to be seen in two different ways: in terms of initial evaluation of the certificate (before correction) and at the end of the entire process.

We first evaluated the initial missing information rate in live birth certificates in January 2008 (baseline situation) and the corresponding rate at the end of 2008 after complete information had been given to the city civil servants and midwives and gynaecologists. The data were evaluated again at the end of 2009 where no reinforcement rules were adopted. The residual missing data rates after correction in 2008 and 2009 were also compared.

Mother’s origin was defined based on her nationality at birth. Non-Belgian were compared to Belgian natives (34.8%), and six immigrant subgroups were identified and compared to Belgian natives: Eastern Europe (8.9% of all births): Albania, Bulgaria, Czech Republic, Hungary, Poland, Romania, Slovakia, the former Soviet republics, and the former Yugoslavia; Former 15-European Union member countries (12.5%); Maghreb (22.5%): Algeria, Morocco, and Tunisia; South-East Asia (2.4%); Sub-Saharan Africa (9.3%); Turkey (3.9%). Stillbirths’ certificates are encoded in another regional Centre, and the completeness of certificates was therefore only assessed in live births.

The stillbirth rate for deliveries in Brussels in 2007 was 4.7 ‰ (110/23170) (unpublished data), from which 22.8% were <28 weeks (baseline situation) [12]. Stillbirth rates were calculated in 2008 and 2009 and compared with the previous data.

Multiple gestations rates were calculated in 2008 and 2009.

Differences between groups were compared by chi-square analyses. A significance level of 0.05 was used in all statistical tests. Statistical calculations were undertaken using the STATA software (version 10.0, College Station, Texas, USA).

## Results

### Missing information

Total initial missing information and suspected inconsistencies sent back to maternities and city civil servants initially accounted for 64.0% (1826 out of 2852 certificates received) and 20.8% (728/3506) and 19.5% (544/2792) in January 2008 and December 2008 and 2009 respectively (*p*-value = 0.22 between December 2008 and 2009). Denominators are not strictly similar due to time variations in certificates transmission by all maternities or city offices (e.g., certificates transmitted at the end of January might not comprise all births of January).

Residual missing data rates are summarized in Table 1. After correction with lists sent back to maternities or city offices, the residual missing data rate per variable in maternal and perinatal data was 2.1% in 2008 (range: 0.0-15.5%) and 0.8% (range: 0.0-10.5%) in 2009 (*p*-value < 0.001).

**Table 1:** Residual missing data after correction in Brussels birth certificates in 2008 and 2009

**Variable**	**% missing data in 2008 (N = 23375)**	**% missing data in 2009 (N = 24157)**
**Pregnancy and infant characteristics**		
Date of birth	0.00	0.00
Sex	0.02	0.00
Plurality and birth order	0.00	0.00
Presentation	0.44	0.19
Induction	0.22	0.09
Mode of delivery	0.00	0.12
Gestational age	0.28	0.28
Birthweight	0.18	0.24
Respiratory assistance	15.50	0.37
1 minute Apgar score	0.26	0.35
5 minute Apgar score	0.27	0.36
Transfer to NICU	15.50	0.94
Parity	0.74	0.12
Date of the last delivery	10.15	10.54
Hypertension	0.00	0.00
Diabetes	0.00	0.00
Birth defect	0.03	0.26
Place of delivery	0.00	0.00
Type of maternity	0.00	0.00
**Mother characteristics**		
Birth date	0.05	0.00
Educational level	9.48	6.46
Employment status	1.06	0.90
Type of work	1.45	1.27
Original nationality	2.56	0.02
Present nationality	0.07	0.01
Cohabitation status	0.55	0.26
Marital status	0.15	0.11
Date of marriage	0.40	0.12
Residence	0.09	0.00
**Father characteristics**		
Birth date	1.18	0.71
Educational level	7.57	5.63
Employment status	1.96	1.37
Type of work	1.88	1.43
Original nationality	10.70	0.77
Present nationality	1.44	0.74

*NICU*, Neonatal Intensive Care Unit.

Fathers’ characteristics were also assessed after excluding certificates where the mother declared to live alone and the father was not declared at birth, and the residual missing data rate per variable in paternal, maternal, and infant data was 2.4% in 2008 and 0.9% in 2009 (*p*-value < 0.001).

In women with nationality at birth different than Belgian, some administrative data were significantly more absent in 2008: cohabitation and marital status, maternal educational level, employment status and type of work, as well as father’s characteristics. Medical data were uniformly distributed except for parity, admission to neonatal unit, respiratory assistance, and the date of the last delivery (data not shown). In 2009 differences persisted for the date of the last delivery, maternal educational level, employment status and type of work, and father’s educational level (Table 2). When taking into account specific area of origin, medical and socio-economic characteristics were more frequently missing for women from Sub-Saharan Africa.

**Table 2:** Residual missing data after correction according to maternal origin in Brussels birth certificates in 2009

**Variable**	**% missing data**						
	**Belgian**	**EU15**	**Maghreb**	**Sub-Saharan Africa**	**Eastern Europe**	**Turkey**	**South-East Asia**
**Pregnancy and infant characteristics**							
Transfer to NICU	1.03	0.81	0.79	1.07	0.93	0.82	1.19
Date of the last delivery	**8.12**	**10.87**	**10.71**	**13.02**	**12.35**	**14.38**	**10.32**
**Mother characteristics**							
Educational level	**3.81**	**4.88**	**8.09**	**9.83**	**9.44**	**6.65**	**7.85**
Employment status	**0.52**	**0.90**	**0.98**	**1.32**	**1.19**	**0.72**	**0.51**
Type of work	**0.75**	**1.26**	**1.56**	**1.84**	**1.50**	**1.02**	**1.54**
**Father characteristics**							
Birth date	**0.67**	**0.44**	**0.45**	**1.64**	**0.88**	**0.32**	**1.24**
Educational level	**4.36**	**4.36**	**6.64**	**7.91**	**6.69**	**5.99**	**7.10**
Employment status	**1.15**	**1.07**	**1.21**	**2.49**	**1.56**	**0.95**	**1.42**
Type of work	**1.22**	**1.14**	**1.03**	**3.04**	**1.71**	**0.84**	**1.78**
Original nationality	**0.77**	**0.44**	**0.47**	**1.64**	**1.03**	**0.32**	**1.07**
Present nationality	**0.73**	**0.44**	**0.49**	**1.59**	**0.93**	**0.32**	**1.24**

Analyses were performed if missing rate exceeded 0.5%. Comparison group was Belgian women. In bold: differences statistically significant with a *p*-value <0.001. Not in bold: not statistically significant at a significance level of 0.05.

*NICU*, neonatal Intensive Care Unit; EU15, Former 15-European Union member countries.

### Stillbirths registration

In total, 197 stillbirths were collected in 2008: 8.4 ‰ of all births (ranging from 1.9 to 18.9 ‰ according to the maternity) and 226 in 2009: 9.3 ‰ of all births (range: 4.7- 24.0 ‰), compared to 110 in 2007 (4.7 ‰, *p*-value < 0.001); 50.8% stillbirths were <28 weeks in 2008 and 52.4% in 2009.

### Identification of multiple births

All pairs or triplets have been identified in 2008 and 2009. In some multiple pregnancies, the second birth occurred more than 24 hours after the first birth: 2 in 2008, and 2 in 2009, resulting in different birth data. Only 2 multiple births were reported as comprising one infant and one fetal demise before 22 weeks in 2008 (2/444, 0.5%) as well as in 2009 (2/529, 0.4%).

## Discussion

We have shown that reinforcement of data collection was associated with a decrease of the return rate of correction lists to maternities and city offices and a decrease of the residual missing data rate in live birth certificates.

A previous study had reported reductions in the query rate (the percentage of records with missing or apparently mistaken information that generated queries to hospitals) after introduction of electronic checks [13], and a recent review has suggested that the return rate could be used as a target for evaluation and improvement [1]. The need for a strong relationship between researchers, staff in hospitals, and public health data agencies to support continuous data quality improvement activities has been highlighted before [9,10,14]. Data manager can involve hospitals in recognizing the value of having the data accurately represent the hospital and the population they serve [1]. The Centre is overcoming this by giving an annual individual report to all maternity wards including all reported obstetrical and neonatal events and risk factors related to their population.

The residual missing data rate is low for most variables, as the missing rate of socio-economic variables is above 10% in many European registries [4,15-17]. There were significant improvements in the registration of respiratory assistance and admission to neonatal intensive care unit between 2008 and 2009, which can be explained both by the information provided to health workers and correction lists, as these variables are reported in the delivery file. Information about the date of the last delivery showed no improvement, and a possible explanation is that this information is most of time lacking in the delivery file and it is unlikely that this variable is corrected with means of correction lists. The registration of education was also improved. This improvement is especially due to the information provided to city civil servants, as this variable is obtained from the parents at birth, and is usually not registered by city registration services, limiting the probability of correction by means of correction lists. Conversely, nationality is registered by city registration services and can be obtained by means of correction lists, except in the particular cases of women in illegal residency, for which registration of data at birth is crucial. Missing data rate varies according to immigration status with a clear directional pattern across data elements toward more missing data for immigrant women. Similarly, a Belgian study in the 1980s had found a higher proportion of unknown last menstrual period in perinatal forms from North African mothers [18]. This might indicate difficulties in gathering social data such as information regarding education when schooling has been conducted abroad in a different educational system or language difficulties, and illegal residents might also be less likely to provide information to civil officers. A recent study has observed that underreporting of adverse birth outcomes, risk factors, and obstetric procedures was associated strongly with the lack of English-language proficiency [2]. However Sub-Saharan mothers in Belgium originate mostly from the Democratic Republic of the Congo (DRC), Rwanda, and Burundi, and they have higher rates of missing data despite their usual ability to speak French. This underscores the importance of considering and documenting potential influences on data quality when birth certificate data are used for obstetric and perinatal research. Caution is necessary when discussing both parents’ education level and date of the last delivery in the present series. However, the residual missing data rate is very low.

In Belgium, fetal death reporting is required by law for all pregnancy losses occurring with a fetal weight of at least 500 g or at 22 weeks or more [3]. It has been observed previously that the decision to declare or not to declare rests mainly with the birth attendant, his/her knowledge of the definitions, and, for very preterm births, on his/her confidence in their chances of survival, and social and cultural considerations [3,6], and many fetal deaths may go unreported [5,6,19]. Efforts have been made to inform birth attendants, and the registration seems to be improved compared to 2007.

In a recent twin study where ascertainment was made from the earliest antenatal scan on which a multiple pregnancy was detected, there was loss of a conceptus at 16 weeks gestation in 4.3% and between 16-24 weeks in 0.5% of twin pregnancies, and 4.8% of twin conceptions were thus wrongly classified at delivery as singleton live or stillbirths [20,21]. Our results (0.5% of early loss of a co-twin in 2008 and 0.4% in 2009) are far from these estimates. A recent study comparing birth certificates with hospital discharge abstracts in Flanders has shown slight differences with higher twins rates in discharge abstracts (2.0 versus 1.6% in 2004), suggesting that the latter could be more complete regarding early losses [22]. Financial incentives could prompt hospitals to maximize the coding of complications, although it is not possible to know the extent of these differences [1,22]. Fetal death in a multiple gestation has clinical implications for a surviving co-conceptus, such as preterm birth and cerebral impairment, and failure to register an early death in a multiple gestation may have statistical repercussions [20,21].

## Conclusions

The findings from this study indicate that reinforcement of data collection was associated with a decrease of the initial missing information and the residual missing data rate in live birth certificates. Missing data rate is not uniform across all births and higher for immigrant mothers. However, the residual missing data rate is very low. Fetal death reporting has also been improved when compared to previous data. Finally, the early loss of a co-twin before 22 weeks seems to remain underreported.

Additional studies are necessary to further explore missing information findings in the stillbirth certificates, and a long-term evaluation of the registration of stillbirths is needed.

## Competing interests

The authors declare that they have no competing interests.

## Authors’ contributions

AFM participated in gathering and correcting the database and drafted the manuscript. PB participated in the design of the study and helped to draft the manuscript. MD participated in gathering and correcting the database and revising the manuscript. VVL participated in gathering and correcting the database and revising the manuscript. SVD participated in gathering and correcting the database and revising the manuscript. YE participated in the design of the study and participated in gathering and correcting the database and helped to draft the manuscript. All authors read and approved the final manuscript.
